# The COMBAT project: controlling and progressively minimizing the burden of vector-borne animal trypanosomosis in Africa

**DOI:** 10.12688/openreseurope.14759.2

**Published:** 2022-08-15

**Authors:** Alain Boulangé, Veerle Lejon, David Berthier, Sophie Thévenon, Geoffrey Gimonneau, Marc Desquesnes, Samuel Abah, Prudenciène Agboho, Kalinga Chilongo, Tsegaye Gebre, Assane Gueye Fall, Dramane Kaba, Stefan Magez, Daniel Masiga, Enock Matovu, Aldjibert Moukhtar, Luis Neves, Pamela A. Olet, Soumaïla Pagabeleguem, William Shereni, Brice Sorli, Moeti O. Taioe, María Teresa Tejedor Junco, Rehab Yagi, Philippe Solano, Giuliano Cecchi

**Affiliations:** 1 CIRAD, UMR INTERTRYP, Bouaké, 01 BP 1500, Cote d'Ivoire; 2 CIRAD, IRD, INTERTRYP, Univ of Montpellier, Montpellier, F-34398, France; 3 CIRAD, IRD, UMR INTERTRYP, Univ of Montpellier, Montpellier, F-34398, France; 4 CIRAD, UMR INTERTRYP, Montpellier, F-34398, France; 5 CIRAD, UMR INTERTRYP, Dakar-Hann, BP 2057, Senegal; 6 CIRAD, UMR INTERTRYP, Toulouse, F-31076, France; 7 Mission Spéciale D'Eradication des Glossines (MSEG), Ministère de l'Elevage, des Pêches et des Industries Animales, Ngaoundéré, BP 263, Cameroon; 8 Centre International de Recherche-Développement sur l’Elevage en zone Subhumide (CIRDES), Bobo-Dioulasso, 01 BP 454, Burkina Faso; 9 Tsetse and Trypanosomosis Control Unit (TTCU), Ministry of Fisheries and Livestock, P.O Box 50197, Lusaka, 10101, Zambia; 10 National Institute for Control and Eradication of Tsetse and Trypanosomosis (NICETT), P.O Box 19917, Addis Ababa, Ethiopia; 11 Institut Sénégalais de Recherches Agricoles (ISRA), Dakar-Hann, BP 2057, Senegal; 12 Institut Pierre Richet (IPR), Institut National de Santé Publique, Bouaké, 01 BP 1500, Cote d'Ivoire; 13 Laboratory of Cellular and Molecular Immunology, Vrije Universiteit Brussel (VUB), Brussels, B-1050, Belgium; 14 International Centre of Insect Physiology and Ecology (ICIPE), Nairobi, 00100, Kenya; 15 Makerere University, Kampala, 10218, Uganda; 16 Institut de Recherche en Elevage pour le Développement (IRED), N'Djamena, Route de Farcha, BP 433, Chad; 17 Centro de Biotecnologia, Universidade Eduardo Mondlane, Maputo, 00200, Mozambique; 18 Department of Veterinary Tropical Diseases, Faculty of Veterinary Sciences, University of Pretoria, Onderstepoort, 0110, South Africa; 19 Kenya Tsetse and Trypanosomosis Eradication Council (KENTTEC), Nairobi, 00800, Kenya; 20 Insectarium de Bobo-Dioulasso – Campagne d'Eradication de la mouche Tsé-tsé et de la Trypanosomose (IBD-CETT), Ministère des ressources animales et halieutiques, Bobo-Dioulasso, 01 BP 1087, Burkina Faso; 21 Division of Tsetse Control Services (TCD), Ministry of Lands, Agriculture, Fisheries, Water and Rural Development, P.O Box CY52, Harare, Zimbabwe; 22 Institut d'Electronique et des Systèmes (IES), Université de Montpellier, Montpellier, F-34090, France; 23 Onderstepoort Veterinary Research, Agricultural Research Council (ARC), Pretoria, 0110, South Africa; 24 Universidad de Las Palmas de Gran Canaria (ULPGC), Las Palmas de Gran Canaria, 35016, Spain; 25 Central Veterinary Research Laboratory (CVRL), Animal Resources Research Corporation, Khartoum, 12217, Sudan; 26 Animal Production and Health Division, Food and Agriculture Organization of the United Nations (FAO), Rome, 00153, Italy

**Keywords:** Trypanosomosis, nagana, surra, tsetse fly, Stomoxys, Tabanids, trypanotolerance, progressive control pathway

## Abstract

Vector-borne diseases affecting livestock have serious impacts in Africa. Trypanosomosis is caused by parasites transmitted by tsetse flies and other blood-sucking *Diptera*. The animal form of the disease is a scourge for African livestock keepers, is already present in Latin America and Asia, and has the potential to spread further. A human form of the disease also exists, known as human African trypanosomosis or sleeping sickness. Controlling and progressively minimizing the burden of animal trypanosomosis (COMBAT) is a four-year research and innovation project funded by the European Commission, whose ultimate goal is to reduce the burden of animal trypanosomosis (AT) in Africa. The project builds on the progressive control pathway (PCP), a risk-based, step-wise approach to disease reduction or elimination. COMBAT will strengthen AT control and prevention by improving basic knowledge of AT, developing innovative control tools, reinforcing surveillance, rationalizing control strategies, building capacity, and raising awareness. Knowledge gaps on disease epidemiology, vector ecology and competence, and biological aspects of trypanotolerant livestock will be addressed. Environmentally friendly vector control technologies and more effective and adapted diagnostic tools will be developed. Surveillance will be enhanced by developing information systems, strengthening reporting, and mapping and modelling disease risk in Africa and beyond. The socio-economic burden of AT will be assessed at a range of geographical scales. Guidelines for the PCP and harmonized national control strategies and roadmaps will be developed. Gender equality and ethics will be pivotal in all project activities. The COMBAT project benefits from the expertise of African and European research institutions, national veterinary authorities, and international organizations. The project consortium comprises 21 participants, including a geographically balanced representation from 13 African countries, and it will engage a larger number of AT-affected countries through regional initiatives.

## Disclaimer

The views expressed in this article are those of the authors. Publication in Open Research Europe does not imply endorsement of the European Commission.

## Background

### The European Commission call for proposals on vector-borne diseases in Africa

Agriculture in the 21 ^st^ century faces the challenge of sustainable production. Arguably more than other continents, Africa needs to secure adequate food production ( FAO *et al*., 2020) while facing climate change, which can increase the risk of emergence and spread of vector-borne infections. Not only do such diseases affect animals and reduce livestock production, but, in the case of zoonoses, they can be transmitted to humans as well. Furthermore, globalisation increases the risk of pathogens spreading outside their original areas of endemicity. If disease control is to be improved, a better understanding of these vector-borne infections is needed, including their transmission cycles, vectors and potential for spread.

Against this background, in 2019 the European Commission launched a call for projects to improve knowledge on vector-borne diseases impacting on animal husbandry in Africa [SFS-35-2019-2020 (sub-topic C): Sustainable intensification in Africa; Scope C: Vector-borne diseases in Africa. https://ec.europa.eu/info/funding-tenders)]. The focus was on infections that seriously affect the African continent and that could pose a threat to Europe. Proposals were required to address: (i) vector competence and ecology, and vector-pathogen interactions; (ii) the association between host immunity, pre-existing immunity, immunization and pathogen distribution; (iii) vulnerability to infection of different livestock species and breeds, (iv) new diagnostics targeting either antibodies or pathogens; (v) tools for disease prevention; (vi) risk mapping, including the risk of spread; (vii) assessment of the socio-economic burden of the disease; and (viii) monitoring tools and reinforced surveillance.

Projects were expected to promote prevention, control and minimization of vector-transmitted animal diseases. More specific expected impacts included the delivery of performant diagnostics suited for field use, reinforcement of disease surveillance for improved knowledge of incidence and impacts, enhanced estimation of the risk for geographical expansion of the disease, appropriate estimation of disease burden, rationalization and improved targeting of disease control.

In response to the call, the project “Controlling and progressively Minimizing the Burden of Animal Trypanosomosis” (COMBAT) was developed ( https://cordis.europa.eu/project/id/101000467).

### Vector-borne animal trypanosomosis

Vector-borne animal trypanosomosis (AT) is a group of diseases caused by various unicellular protozoan parasites ( *Trypanosoma* spp.). In its different forms, AT occurs in virtually all countries in Africa. *Trypanosoma brucei*, *T. congolense, T. vivax* and *T. simiae* are transmitted cyclically by tsetse flies ( *Glossina* spp.), causing the form of the disease called ‘nagana’ ( Diall *et al*., 2017; OIE, 2021). *T. vivax* can also be transmitted mechanically by other biting flies such as stable flies ( *Stomoxys*) and horseflies (Tabanids), which have enabled it to spread to Latin America ( Gonzatti *et al*., 2014) and, as recently reported, Asia ( Asghari & Rassouli, 2022). These other biting flies also transmit *T. evansi*, the causative agent of ‘surra’, a disease that is endemic in large parts of Africa, Asia and Latin America, and also present in the Canary Islands (Spain) ( Aregawi *et al*., 2019). Surra incursions have been reported in continental Europe ( Gutierrez *et al*., 2010) but, despite the presence of competent vectors, establishment and further spread have so far been avoided thanks to an effective response and reinforced surveillance ( Desquesnes *et al*., 2009; Tamarit *et al*., 2010). Livestock and wild animals can also act as reservoirs for human-infective trypanosomes causing human African trypanosomosis [HAT, also known as ‘sleeping sickness’ ( Büscher *et al*., 2018; Büscher *et al*., 2017)], a deadly neglected tropical disease that caused ravaging epidemics in the 20 ^th^ century. Over 50 million people are at risk of contracting HAT in some twenty countries in Africa ( Franco *et al*., 2022), including tourists and travellers from around the world ( Simarro *et al*., 2012).

In the past twenty years, progress in AT control has been limited. This is in contrast to the great strides made against HAT, which is now targeted for elimination by the World Health Organization (WHO) ( WHO, 2020a). Animal trypanosomosis still kills or reduces the productivity of millions of cattle and other livestock in Africa, while thirty-five million doses of trypanocidal drugs are estimated to be administered every year. Economic losses to livestock producers due to AT are estimated in billions of USD, with further losses occurring within the agricultural sector as a whole ( Shaw *et al*., 2017).

There are many reasons why AT continues to afflict African livestock keepers and hamper food security ( Diall *et al*., 2017). First, the disease is complex, with several species of hosts, parasites, and vectors, a reservoir of infections in wildlife, and a range of unanswered epidemiological questions. Second, existing control tools are not up to the challenge, with no vaccine, sub-optimal vector control tools, no cost-effective pen-side diagnostic, and long outdated drugs associated with the problems of counterfeiting and drug resistance. Third, AT control is hampered by weak surveillance systems, insufficient socio-economic data on its impact, and poor strategic planning. In addition, farmers and veterinary services have limited access to state-of-the-art technology, and, when technology is available, they often lack the expertise to apply it effectively. Finally, and crucially, most AT-affected countries are low-income countries, with limited resources to control endemic livestock diseases, and a low level of awareness of decision-makers, donors and national veterinary authorities.

## The Project COMBAT

To have a lasting, continent-wide impact on the burden of AT, the COMBAT project will implement a series of coordinated activities tackling a wide range of challenges: acquisition of epidemiological knowledge to inform decision-makers, development of innovative tools, risk mapping and modelling, socio-economic assessments, capacity building, advocacy, and stakeholders’ engagement. The project will also develop internationally agreed guidelines for the progressive control of AT and streamline them into national strategies and policies.

COMBAT uses the Progressive Control Pathway (PCP) for AT as its overarching strategic framework (see Box), and the project will enable international organizations, veterinary authorities and researchers to fully develop and apply the approach.

**Box: The progressive control pathway for AT**

Scarcity of rational, evidence-based strategies is one of the obstacles to sustainable AT control. It is to remove this hurdle that the Food and Agriculture Organization of the United Nations (FAO) and its partners promoted the development of the progressive control pathway (PCP) for AT ( Diall *et al*., 2017). Progressive control pathways are risk-based approaches to structure the road to disease reduction or freedom through a series of achievable steps. They are widely recognized as effective conceptual frameworks to tackle a number of diseases of animals and humans (e.g. foot-and-mouth-disease ( Sumption *et al*., 2012), *peste des petits ruminants* (PPR), rabies, brucellosis). They are also increasingly applied in other domains such as fisheries and anti-microbial resistance. Notably, PCPs are used by a broad range of national health authorities and international stakeholders [e.g. FAO, World Organisation for Animal Health (OIE), World Health Organization (WHO), International Atomic Energy Agency (IAEA), and African Union (AU)].

The PCP for AT includes five stages. The pre-entry level (below stage 1) emphasizes political and institutional commitment. Stage 1 focuses on mapping disease risk and impacts, development of capacities and strategies, prioritization of intervention areas and pilot control activities. Stage 2 aims at sustainable and economically profitable reduction in AT. Stages 3 to 5 target disease elimination, if and where the goal is technically feasible and socio-economically justifiable. Within a country, different areas can be at different stages of the PCP.

While each stage of the PCP focuses on a set of specific activities and objectives, five technical and managerial areas cut across all stages: (i) coordination and stakeholders’ engagement, (ii) establishment and maintenance of an enabling environment, (iii) data collection, management and analysis, (iv) capacity development and (v) AT surveillance and control activities.

While the foundations of the PCP have been laid out ( Diall *et al*., 2017), implementation guidelines are still lacking, and further efforts are needed for the concept to be translated into practice at the country level.

**Figure 1.**
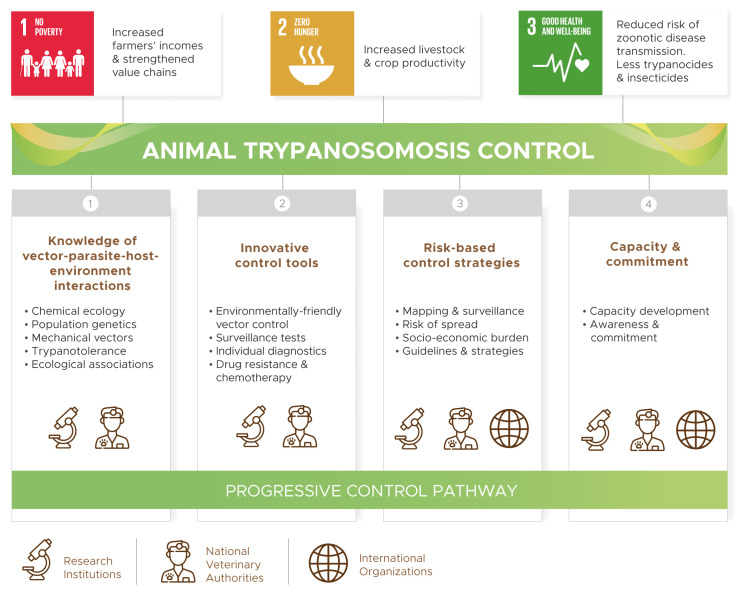
Pillars of the COMBAT project.

The COMBAT project is structured into four thematic pillars ( Figure 1): (1) improved knowledge of AT epidemiology (i.e. vector-parasite-host-environment interactions), (2) development of innovative control tools, (3) disease risk mapping, surveillance and evidence-based control strategies, and (4) reinforced capacities and engagement of stakeholders. Data collection, analysis and storage for evidence-based decision making underpin all COMBAT activities.

### Improved knowledge of vector-parasite-host-environment interactions (pillar 1)

Important gaps remain in our knowledge of the actors of AT epidemiological cycles, that is, vectors, parasites and their hosts, livestock and wildlife alike, and the environment. Tsetse flies have been extensively studied, but the ecological picture is changing rapidly because of population growth, climate and land cover change. On the other hand, little is known about the biology and competence of mechanical vectors, although they pose a high risk for disease spread. Furthermore, the mechanisms and genetic underpinnings of trypanotolerance are poorly known, even though tolerant breeds can be a viable option for livestock rearing in enzootic areas while also contributing to the conservation of biodiversity. Regarding trypanocidal drug resistance, its molecular mechanisms still need to be elucidated. Through fundamental and applied research using cutting-edge technology, COMBAT will improve knowledge of the interactions between key players of AT in their environment.

***Chemical ecology of AT vectors.*** In tsetse flies, studies on chemical ecology have mainly focused on host-vector interactions ( Gibson & Torr, 1999), e.g. chemical attractants mimicking the odour of hosts to enhance the efficiency of traps and targets ( Rayaisse *et al*., 2010), or olfactory repellents to develop protective devices ( Saini *et al*., 2017). However, to date there are no tsetse-control tools targeting gravid females, which could remove adult flies as well as their larvae. Also, mating and larviposition behaviours are governed by a combination of olfactory and visual stimuli ( Gimonneau *et al*., 2021). The COMBAT project will identify and evaluate the chemicals used by tsetse flies for mating and larviposition, which could offer opportunities for behavioural manipulations and the development of highly specific vector control tools targeting males and gravid females.

In *Stomoxys*, oviposition is mediated by compounds that induce oviposition in specific sites or substrates ( Baleba *et al*., 2020) and mating behaviour is mediated by female attractants that lure male insects. Some compounds have been tested with promising results in the field to boost trap efficiency and others are still to be identified ( Baleba *et al*., 2019). COMBAT will optimize the use of these semiochemicals for the control of stable flies, including a suitable formulation for gradual release.

***Tsetse population genetics.*** Population genetics is an effective tool to assess whether a given tsetse population is isolated from neighbouring ones. Given the risk of reinvasion, information on the level of isolation is crucial to establish whether local tsetse elimination, also referred to as ‘local eradication’, could be a suitable strategy in a potential intervention area ( Bouyer *et al*., 2015). The COMBAT project will assess tsetse isolation by measuring gene flow in selected areas where elimination is considered ( Solano *et al*., 2010).

In an area of Côte d'Ivoire, a selection signature has been observed at a microsatellite allele during a tsetse control campaign ( Berté *et al*., 2019). This suggests that, through an unknown mechanism, tsetse control may select for the emergence of resistant flies. COMBAT will further investigate this phenomenon.

Finally, when the sterile insect technique (SIT) is used, the release of sterile male tsetse needs to be sustained until all wild flies have been eliminated ( Vreysen *et al*., 2000). In this kind of vector control operation, entomological surveys allow the evaluation of survival and competitiveness of sterile males. This is normally done by marking sterile male flies with a fluorescent dye, an imperfect technique because of the possibility of insufficient marking or because of dye contamination between sterile and wild flies in the traps. COMBAT will further develop genetic markers to differentiate more accurately wild from sterile male flies of the same species ( Pagabeleguem *et al*., 2016).

***Ecology and vector competence of mechanical vectors of AT.*** Tabanids and *Stomoxys* are cosmopolitan hematophagous flies ( Baldacchino *et al*., 2018) that act as mechanical vectors of blood pathogens. Because of interrupted feeding, and since their infectivity is short-lived, tabanids can cause ‘intra-herd transmission’. In *Stomoxys*, the ingested blood can be directed towards the gut or towards the crop, and survival of trypanosomes in the crop allows transmission at one- to three-day interval ( Foil & Hogsette, 1994), with the possibility of ‘inter-herd transmission’. The phenomenon has been described in experimental conditions ( Baldacchino *et al*., 2013), but investigations are needed to determine vector competence. COMBAT will determine the conditions for delayed mechanical transmission, i.e. directing blood towards the crop, blood volume, the trypanosomes survival time in the crop, and regurgitation using laboratory reared *Stomoxys* and rodents experimentally infected by *T. evansi* as a model. This knowledge will be used to tackle trypanosome transmission in the presence of *Stomoxys*.

***Livestock genetic diversity and trypanotolerance.*** The diversity of livestock breeds in Africa is high, and it has been shaped over millennia by human usage and the environment. Animal trypanosomosis also played a role, and a high variability exists in susceptibility to the disease ( Murray *et al*., 1990). West African taurine breeds (e.g. humpless cattle like N’Dama and Lagune) can tolerate the pathogenic effects of AT, hence the term ‘trypanotolerant’. By contrast, zebu (humped cattle), European breeds and their crossbreeds are generally susceptible to AT. Because of this differential susceptibility, a better characterization and exploitation of animal genetic resources could help mitigate the impacts of AT ( Berthier *et al*., 2015; FAO, 2007; Mwai *et al*., 2015; Naessens, 2006). Knowledge of livestock species and breeds has improved in recent years ( Bahbahani *et al*., 2017), but important gaps remain, and the availability of data varies greatly between countries, areas, and livestock species. The COMBAT project will characterize populations of six livestock species (i.e. cattle, sheep, goats, pigs, donkeys and horses), and link them to AT epidemiological data and production systems. Genomic regions harbouring selection signatures will be identified and compared to public data on other livestock populations ( Gautier *et al*., 2009; Mekonnen *et al*., 2019; Serranito *et al*., 2021), quantitative traits loci ( Hanotte *et al*., 2003), and functional information, to propose candidate genes linked to trypanotolerance.

On a related topic, the functional and molecular bases of cattle trypanotolerance still puzzle researchers despite advances in genetics ( Álvarez *et al*., 2016a; Álvarez *et al*., 2016b; Noyes *et al*., 2011), transcriptomics ( Berthier *et al*., 2008; Hill *et al*., 2005) and immunology ( O'Gorman *et al*., 2009; O'Gorman *et al*., 2006; Sileghem *et al*., 1993). While we know that immune response and metabolism are intertwined ( Donnelly & Finlay, 2015), a joint comparative analysis of immunological and metabolic responses to trypanosome infection in tolerant and susceptible breeds is lacking. COMBAT will monitor AT infection and immunological and metabolic responses in N’Dama, the best-known trypanotolerant breed in West-Africa, in susceptible zebu breeds, together with crosses between local breeds and exotic (European) cattle, frequently encountered in peri-urban farms. In Uganda, COMBAT will verify the hypothesised trypanotolerance of a local breed living under high tsetse challenge in the north-eastern part of the country, by comparing its phenotypic features under trypanosome infection with the more common Ankole cattle, known for its susceptibility.

Another important gap in our knowledge concerns the infectious potential for tsetse flies of susceptible versus trypanotolerant cattle ( Moloo *et al*., 1999), and whether the latter can act as a reservoir for infection. COMBAT will explore the role of trypanotolerant versus trypanosusceptible livestock in the epidemiology of AT by testing whether tsetse flies have the same probability to acquire trypanosomes when feeding on experimentally infected tolerant versus susceptible cattle.

***Ecological associations shaping the transmission cycle.*** Finally, the combined knowledge of a range of epidemiological variables such as vectors’ feeding behaviour, diversity of infecting trypanosomes, favoured ecotypes, and level of insecticide-resistance, can inform AT control. Metabarcoding (i.e. amplicon-based new generation sequencing), starting from the vectors as biological material, can identify these ecological associations, which shape the transmission cycle of vector-borne pathogens ( Hernández-Andrade *et al*., 2019). This approach enabled the transmission cycles of other vector-borne pathogens to be unravelled ( Dumonteil *et al*., 2018), and within COMBAT it will be applied to AT.

### Innovative control tools (pillar 2)

Current tools for the control of AT suffer from a number of shortcomings. COMBAT aims to develop new tools that are performant, appropriately formatted for the settings in which they will be used, and affordable. Environmentally friendly vector control tools will be developed targeting both tsetse and mechanical vectors. Specificity and standardization of diagnostic tools for surveillance at the herd level will be improved for broader adoption at the field level. Tools for the detection of active infections at the individual level allowing subsequent rational chemotherapy will be developed following the REASSURED criteria (i.e. Real-time connectivity, Ease of specimen collection, Affordability, Sensitivity, Specificity, User-friendliness, Rapidity and robustness, be Equipment free, and Deliverable to end-users) ( Land *et al*., 2019). A new treatment with potential to clear infections with trypanosomes that are refractory to the existing drugs will be tested ( Akama *et al*., 2018).

***Vector control and surveillance.*** The main tools for the control of tsetse flies are insecticide treated cattle and attracting devices made, notably, of blue and/or black fabric. The latter can either capture tsetse (i.e. traps, without insecticide) or kill them (i.e. targets, impregnated with insecticide). Traps come in different designs, but they all rely on visual and/or olfactory cues to attract the flies ( Torr & Solano, 2010). Targets are simplified pieces of the same attractive cloth, which are impregnated with insecticide, normally synthetic pyrethroids. Targets, and in particular tiny targets, are also used to support the elimination of gambiense HAT ( FAO & WHO, 2022). Unfortunately, the availability of these tools against tsetse is limited in the rural areas where they are most needed, and the development of traps and targets for mechanical vectors such as *Stomoxys* spp. and *Tabanus* spp. has been neglected. However, new promising polyethylene targets have been recently developed for the combined control of tsetse, tabanids and *Stomoxys* spp. ( Desquesnes *et al*., 2021). The COMBAT project will improve and validate new models of traps and targets that are cheaper, user-friendly, robust, more environmentally friendly and effective against both tsetse and mechanical vectors. COMBAT will also develop new insecticide-free, biodegradable devices.

In addition to stationary baits (i.e. traps and targets), insecticide-treated cattle represent an important tool to control biting flies and ticks, as animals act as mobile baits. However, direct insecticide application on livestock can lead to contamination of animal products, and thus poses hazards to human and environmental health ( Mann *et al*., 2015; Vale *et al*., 2004). COMBAT will test ecologically sound plant extracts for their insecticidal or repellent activity against tsetse flies, including their mode of application on cattle and the duration of activity.

COMBAT will also work on vector surveillance tools. Trapping tsetse flies for surveys is laborious, costly and may be risky for field workers, particularly in conservation areas. Drones have previously been used to monitor and/or control different insect species, and they have also been used for the release of sterile tsetse flies in the context of SIT. COMBAT will attempt to customize drones for tsetse trapping.

Another challenge in vector surveillance is detecting the presence of tsetse when their population densities are very low, as would be expected in the event of a successful tsetse control campaign. In these contexts, exposure of cattle to tsetse can be established from the presence of tsetse saliva antibodies ( Somda *et al*., 2016). However, native whole saliva antigens present several limitations, including mass production, reproducibility, specificity and sensitivity. COMBAT will explore replacement of whole saliva antigens by synthetic peptides.

***AT diagnosis and epidemiological surveillance.*** There exist several diagnostic tests for AT, but they all have their shortcomings.

Parasitological tests, which are currently the most widely used, have low sensitivity, particularly in the chronic phase of the disease. They are also time-consuming and ill-adapted to individual diagnosis ( Desquesnes *et al*., 2022). DNA detection techniques are more sensitive, but the cost and the need for trained technicians and a laboratory environment limit their application ( Büscher & Deborggraeve, 2015). Even simplified formats such as loop-mediated isothermal amplification (LAMP) and recombinase polymerase amplification lateral flow assays ( Li *et al*., 2020), remain too technical for large scale application.

As to the detection of trypanosome-specific antibodies, the present antibody-ELISA tests mainly rely on whole parasite lysates as antigens, and they are useful for AT surveillance, including for monitoring control campaigns ( Adam *et al*., 2012; Sow *et al*., 2013). However, in addition to ethical concerns associated with the infection and killing of large numbers of rodents for trypanosomal lysate production, there are issues related to antigen standardisation, stability and test reproducibility. On the one hand, COMBAT will standardize the antibody-ELISA test through antigen produced from parasites grown *in vitro*, followed by lyophilisation for stabilisation. The inclusion of standardized sera as internal stabilised controls will ensure reproducibility. Furthermore, tests based on total parasite proteins as antigen are usually sensitive but may lack specificity. By contrast, the use of recombinant proteins tends to increase specificity, at the cost of sensitivity, but allows standardisation. COMBAT will therefore also exploit this principle and investigate the use of a combination of several recombinant proteins, either as mixture or in the form of genetically engineered chimeras.

Contrary to antibody detection, which can indicate either past or present contact with trypanosomes, detection of trypanosome antigens identifies active infection, and can inform treatment decisions. The principle of the detection of parasite products in the host fluids remains a valid proposition, even though the antigen detection ELISAs for AT developed in the past had unsatisfactory diagnostic performances ( Eisler *et al*., 1998). COMBAT will develop two innovations in the detection of trypanosome antigens: i) the capture of a *T. congolense* target enzyme, followed by detection through its enzymatic activity; and ii) the coupling of nanobodies with a radio frequency identification (RFID) sensor format. Both are well suited to adaptation into a rapid diagnostic format for point-of-care diagnosis.

***Rhodesiense human African trypanosomosis.*** Two subspecies of *T. brucei* cause HAT: *T. b. gambiense* and *T. b. rhodesiense* ( Büscher *et al*., 2017). The rhodesiense form of the disease is a zoonosis, with reservoirs in both domestic and wild animals ( WHO, 2013). Screening tests for gambiense HAT exist, and they detect specific antibodies. Seropositive individuals are subsequently examined microscopically, and those confirmed are treated ( WHO, 2013). For rhodesiense HAT, no simple screening test exists, and infections are mostly detected during microscopic examination of blood smears for malaria. However, malaria rapid diagnostic tests are increasingly replacing microscopy, with negative impacts on the detection of rhodesiense HAT ( Franco *et al*., 2020). Because of this, WHO considers the development of a screening test for rhodesiense HAT a priority ( WHO, 2020b). COMBAT will develop antigen detection tests that are *Trypanozoon* specific ( *T. evansi* + *T. brucei* ssp.), which can be used to detect *T. b. rhodesiense* in humans.

***Chemotherapy and drug resistance.*** The two main drugs that are used for the treatment or prophylaxis of AT in cattle are diminazene aceturate and isometamidium chloride ( Giordani *et al*., 2016). However, these compounds have been extensively used, often misused, and frequently counterfeited for decades, which caused the emergence of drug resistance. The problem has been reported from more than 20 countries. Still, the exact gene or genes that are responsible for resistance are not known, which hinders the development of a molecular detection test ( Delespaux *et al*., 2006; Munday *et al*., 2013). COMBAT will collect trypanosomes, perform full genome sequencing and annotation of both resistant and susceptible isolates, followed by comparative analysis. The genes or gene mutations responsible for resistance to trypanocides will be identified and analysed in an effort to identify diagnostic genetic markers for single or multiple resistance. Efforts will focus chiefly on *T. congolense* from West, East and Southern Africa, and to a lesser extent on *T. evansi* from Sudan, while other trypanosomes species may be considered.

Developing new, better treatments against AT would also greatly contribute towards control of the disease and curb drug resistance. Compounds in the benzoxaborole family act by inhibiting the parasite’s mRNA processing ( Wall *et al*., 2018), and these compounds show high *in vitro* activity against *T. congolense, T. vivax, T. brucei* and *T. evansi* ( Akama *et al*., 2018; Ding *et al*., 2010). COMBAT will examine the efficacy of this class of compounds against resistant strains *in vivo*.

### Risk-based control strategies (pillar 3)

Decision-making in the progressive control of AT must be based on the assessment of the risk and impact of the disease, and on rational control strategies. In particular, there is a need for improved mapping of AT and its vectors, reinforced surveillance, and up-to-date estimations of the socio-economic disease burden and the risk of spread. Based on this evidence, strategies for disease control at the national level can be developed, which would benefit from harmonized international guidelines. All these activities will be tackled by COMBAT.

***Disease mapping, surveillance systems and risk of spread.*** Despite the vast amount of data collected in the field over the years, Africa-wide AT maps are absent and continental tsetse maps are long out-of-date ( Ford & Katondo, 1977). Comprehensive national-level datasets are also lacking in most countries. To address these gaps, the continental atlas of tsetse and AT is being developed ( Cecchi *et al*., 2015; Cecchi *et al*., 2014; de Gier *et al*., 2020), and the adaptation and uptake of the methodology at the national level is being promoted in several countries ( Ahmed *et al*., 2016; Diarra *et al*., 2019; Ngari *et al*., 2020; Percoma *et al*., 2022; Shereni *et al*., 2021). COMBAT will complete the development of the continental atlas, extend it to surra ( *T. evansi*), and develop, enhance or update the national atlases in project countries.

Disease surveillance and reporting is weak in many AT-affected countries. In particular, reporting is often patchy, slow, and still relies on hand-written hard copy recording sheets. Data entry errors are frequent and quality control is limited. Furthermore, AT surveillance may be totally absent in certain areas, especially beyond the tsetse belt. COMBAT will strengthen surveillance by identifying and filling gaps in its geographical coverage, reinforcing capacities for data collection, harmonizing reporting formats, promoting quality control protocols and strengthening data management and digitization. The use of information technology, including mobile communications, Global Positioning System (GPS) and Geographic Information System (GIS) will be broadened for speedier, error-free reporting. Stakeholders, including field actors, will also be engaged and sensitized to AT surveillance.

Animal trypanosomosis and its vectors may already be present in many areas in Africa where surveillance is poor or non-existent, and global change could also enable them to spread into currently unaffected areas. Tsetse may also have disappeared from previously-infested areas ( Courtin *et al*., 2010). In Europe, mechanically-transmitted AT has been present in the Canary Islands (Spain) for many years with the *T. evansi* form (surra) ( Gutierrez *et al*., 2000), and incursions in mainland Europe have been reported ( Gutierrez *et al*., 2010). *Trypanosoma vivax*, which spread to Latin America long ago, was recently reported from Asia for the first time ( Asghari & Rassouli, 2022), and potentially-competent mechanical vectors are widely present in Europe. COMBAT will use ecological-niche modelling and up-to-date, comprehensive field data from atlases to predict where AT and its vectors may be occurring undetected within African countries, and where they may spread in the future. The risk of entry and exposure in continental Europe will be assessed with a view to informing risk mitigation.

***Socio-economic burden of AT.*** Estimates of the AT burden in Africa are outdated and based on sparse data ( Kristjanson *et al*., 1999). Knowledge at the national and sub-national level is also poor. This constrains cost-effective targeting of control activities and weakens the commitment of decision makers. COMBAT will assess the burden of the disease at the continental level and, in selected countries, at the national and local level. These assessments will benefit from comprehensive disease datasets developed by the project (i.e. the atlases). Estimates of the burden of AT will inform strategic decisions on where disease control is most needed and provide a baseline to measure progress in disease control. Furthermore, COMBAT will investigate whether vector control activities against gambiense HAT in two countries, Chad ( Rayaisse *et al*., 2020) and Côte d’Ivoire ( Kaba *et al*., 2021), have had positive impacts on AT, in an effort to document the One-Health benefits of trypanosomosis control.

***Guidelines for the progressive control pathway and national strategies.*** Decision-making in AT control requires complex choices to be made in relation to goals, strategies, tactics, tools, and stakeholders’ priorities. These choices carry important technical and financial implications, and they ultimately determine the level of success, cost-effectiveness and sustainability of the interventions. The PCP approach was adapted to AT to help tackle these strategic challenges ( Diall *et al*., 2017), but implementation guidelines are still lacking. Furthermore, and crucially, veterinary authorities have yet to operationalize the approach at the national level. COMBAT will generate internationally agreed guidelines for the PCP for AT, and it will improve awareness and knowledge of the approach. At the country level, the project will promote the development of PCP-smart national strategies and the related implementation roadmaps.

### Technical capacities and institutional commitment (pillar 4)

Livestock keepers, veterinary services and AT control programmes often use outdated or suboptimal tools, have limited access to state-of-the-art technology and need more opportunities for training and capacity development. Furthermore, insufficient resources are allocated to AT control because of the low level of awareness of decision-makers, donors and national authorities. Within COMBAT, the development of knowledge, tools and strategies will be accompanied by extensive capacity building, advocacy and awareness raising at all levels, in order to have a real and lasting impact on disease control in the field.

***Capacity development.*** Enhanced capacities for AT control are crucial to achieve COMBAT’s innovation goals and its desired impacts. A range of training, technology transfer and capacity development activities are planned. Beneficiaries will include farmers, veterinary practitioners, extension workers, students and researchers. Emphasis will be placed on the transfer of state-of-the-art technologies, as well as on the new, innovative tools developed by the project. These include, but are not limited to, genotyping, population genetics, metabarcoding, trapping and identification of AT vectors, AT diagnosis, bioinformatics, data analysis, GPS/GIS, disease risk mapping, and economics. Traditional and virtual training courses will be combined with workshops, exchange visits, mentorship, programmes for advanced academic qualifications (e.g. MSc, PhD, etc.), and on-the-job training. More than one thousand people will be directly reached by COMBAT’s capacity development activities, thus greatly broadening the pool of experts on AT control in Africa.

***Awareness and commitment.*** Despite its heavy burden on African agricultural productivity, AT is often neglected by decision-makers. Reasons for this are manifold ( Roger *et al*., 2017). Animal trypanosomosis hits poor livestock keepers the most, and mainly in rural, remote areas. The disease can also be underestimated by the affected communities themselves, because of a lack of knowledge and poor access to diagnostic tools and veterinary assistance. Finally, despite the recorded incursions into mainland Europe, the transcontinental risk of AT is often overlooked. COMBAT will increase awareness of AT impacts across the board. The reassessment of the burden of AT will generate evidence and revive the interest of donors. The direct involvement of FAO, the engagement of AU and the external support of OIE, WHO and IAEA, will ensure the commitment of the leading international organizations. The strong participation of veterinary authorities in affected countries will draw the attention of national-level decision-makers. Communication and outreach at the grass-roots level will raise the awareness of farmers and extension workers.

### Project consortium and external network

**Figure 2.**
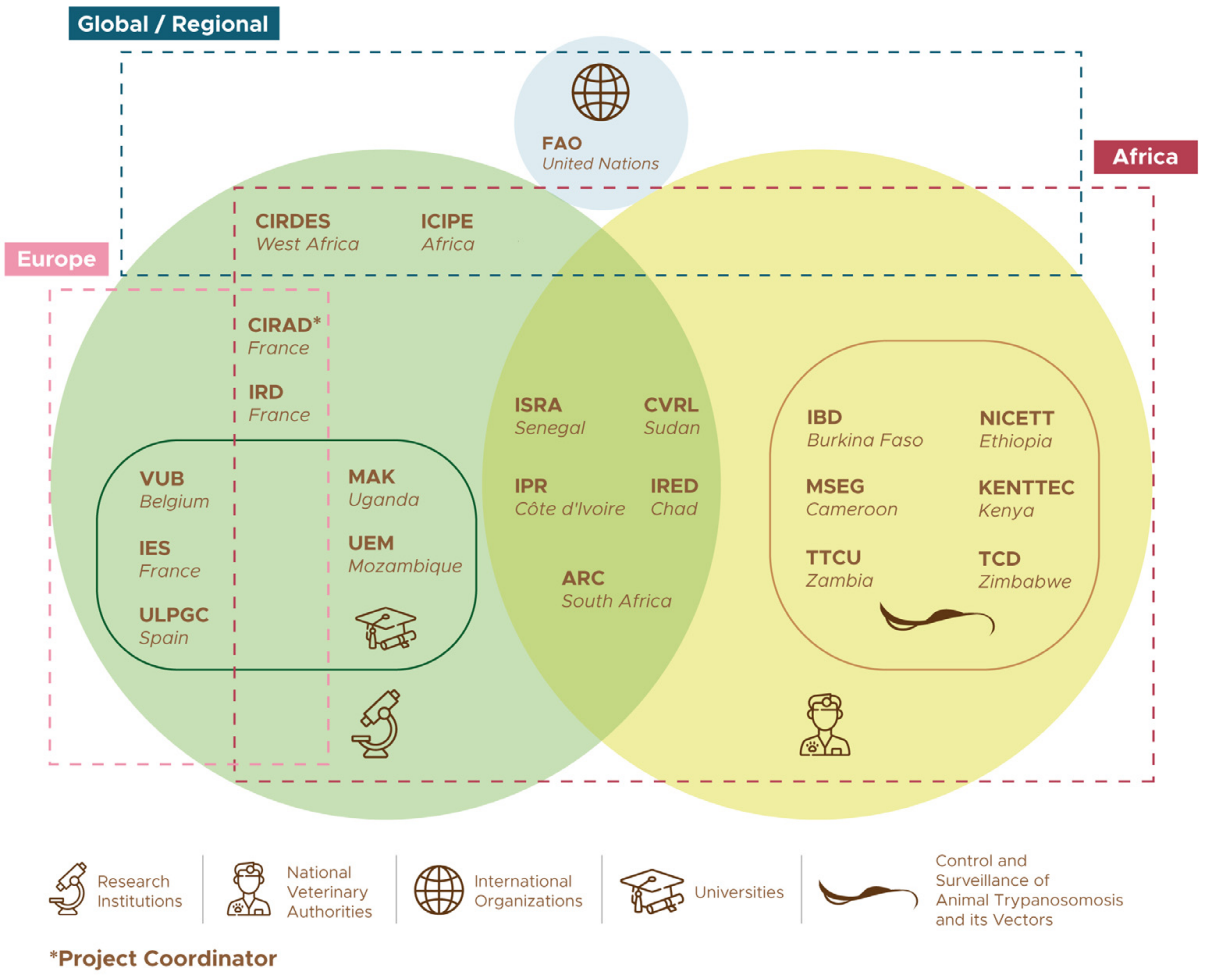
The COMBAT project consortium. The list of acronyms is in Table 1.

***The COMBAT consortium.*** Developing new ways of minimizing the burden of AT, leading to sustainable gains along the PCP, requires technical, organisational and strategic innovation, and it calls for a broad multidisciplinary approach. The COMBAT consortium was designed to mobilize all the necessary expertise to achieve the project’s ambitious objectives. The 21 institutions that participate in the project are listed in Table 1, while Figure 2 provides a schematic representation of their groupings and relationships.

**Table 1.** Participants in the COMBAT project consortium.

Project participant	Acronym	Reference Institution	Country	Type of Institution
*Centre de coopération internationale en * *recherche agronomique pour le développement* *	CIRAD	Ministry for Higher Education, Research and Innovation, Ministry for Europe and Foreign Affairs	France	Research (International development)
*Institut de Recherche pour le Développement*	IRD	Ministry for Higher Education, Research and Innovation, Ministry for Europe and Foreign Affairs	France	Research (International development)
*Institut d'Electronique et des Systèmes*	IES	*Université de Montpellier*	France	Research (University)
*Vrije Universiteit Brussel*	VUB	N/A	Belgium	Research (University)
*Universidad de Las Palmas de Gran Canaria*	ULPGC	N/A	Spain	Research (University)
International Centre of Insect Physiology and Ecology	ICIPE	N/A	Kenya (Africa)	Research (International)
*Centre International de Recherche* *Développement sur l’Elevage en zone Subhumide*	CIRDES	N/A	Burkina Faso (West Africa)	Research (International)
*Universidade Eduardo Mondlane*	UEM	N/A	Mozambique	Research (University)
Makerere University	MAK	N/A	Uganda	Research (University)
*Institut Sénégalais de Recherches Agricoles*	ISRA	*Ministère de l'Agriculture et de* *l’Equipement Rural*	Senegal	Research (National authority)
*Institut Pierre Richet*	IPR	*Institut National de Santé Publique (Ministère * *de la Santé et de l'Hygiène publique)*	Côte d’Ivoire	Research (National authority)
*Institut de recherche en élevage pour le* *développement*	IRED	*Ministère de l'Élevage et des Production* *Animales*	Chad	Research (National authority)
Central Veterinary Research Laboratory	CVRL	Animal Resources Research Corporation (Ministry of Animal Resources and Fisheries)	Sudan	Research (National authority)
Agricultural Research Council	ARC	Department of Agriculture, Forestry and Fisheries	South Africa	Research (National authority)
*Insectarium de Bobo-Dioulasso*	IBD	*Ministère des ressources animales et* *halieutiques*	Burkina Faso	National veterinary authority (tsetse and trypanosomosis)
*Mission Spéciale d'Eradication des Glossines*	MSEG	*Ministère de l'Elevage, des Pêches et des* *Industries Animales*	Cameroon	National veterinary authority (tsetse and trypanosomosis)
National Institute for Control and Eradication of Tsetse fly and Trypanosomosis	NICETT	Ministry of Agriculture	Ethiopia	National veterinary authority (tsetse and trypanosomosis)
Kenya Tsetse and Trypanosomiasis Eradication Council	KENTTEC	Ministry of Agriculture, Livestock, Fisheries and Co-operatives	Kenya	National veterinary authority (tsetse and trypanosomosis)
Tsetse and Trypanosomiasis Control Unit	TTCU	Ministry of Fisheries and Livestock	Zambia	National veterinary authority (tsetse and trypanosomosis)
Division of Tsetse Control Services	TCD	Ministry of Lands, Agriculture, Fisheries, Water, Climate and Rural Development	Zimbabwe	National veterinary authority (tsetse and trypanosomosis)
Food and Agriculture Organization of the United Nations	FAO	N/A	Italy (Global)	International (United Nations)

*Project Coordinator

**Figure 3.**
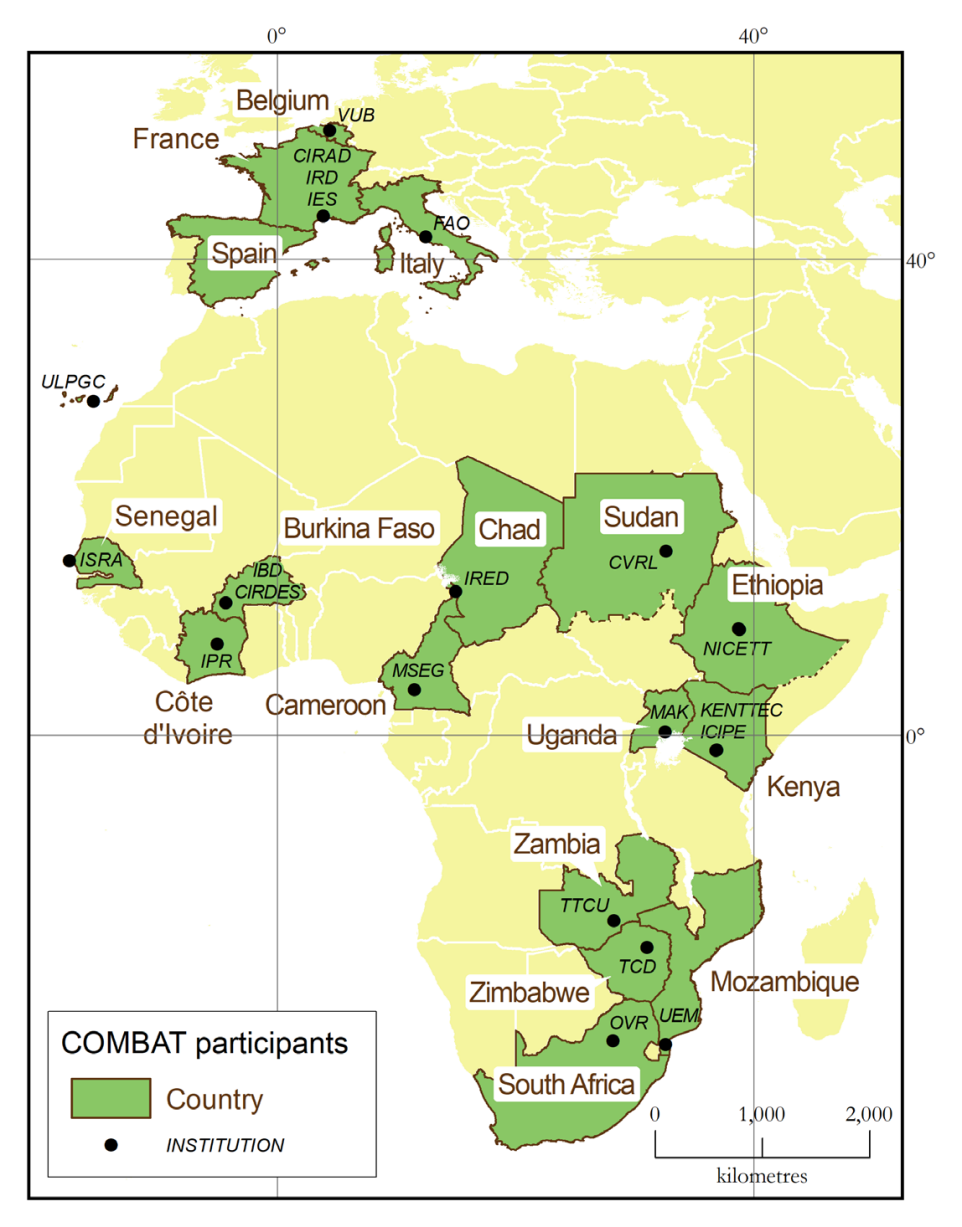
Geographical distribution of COMBAT project participants.

One of the main assets of the COMBAT consortium is the strong representation of AT-affected countries, with a total of 15 African participants from 13 different countries ( Figure 3). This representation from Africa is well-balanced between research institutions (nine) and national authorities in charge of the control and surveillance of AT and its vectors (six). Furthermore, five out of the nine African research institutions are embedded within the national ministries mandated for disease control, thus ensuring a strong linkage with the veterinary services. This deep involvement of national authorities in charge of disease control has few precedents in research projects, and it offers abundant opportunities to maximize the impacts of research.

In addition to the 15 African participants, COMBAT also comprises five European institutions. Two, including the consortium leader (i.e. project coordinator), are the French research institutions mandated to support the development of the Global South (CIRAD and IRD), and they benefit from strong links with, and a long-standing presence in, AT-affected countries. The other three European institutions are universities holding highly specialized expertise, for example in nanobody technology (VUB), RFID sensors (IES), and AT occurrence in Europe (ULPGC).

Finally, the consortium benefits from the involvement of high-profile international organizations. In particular, the FAO is a full-fledged project participant, which with its global network of regional and country offices will help maximize the project’s contribution to the progressive control of AT in Africa.

**Figure 4.**
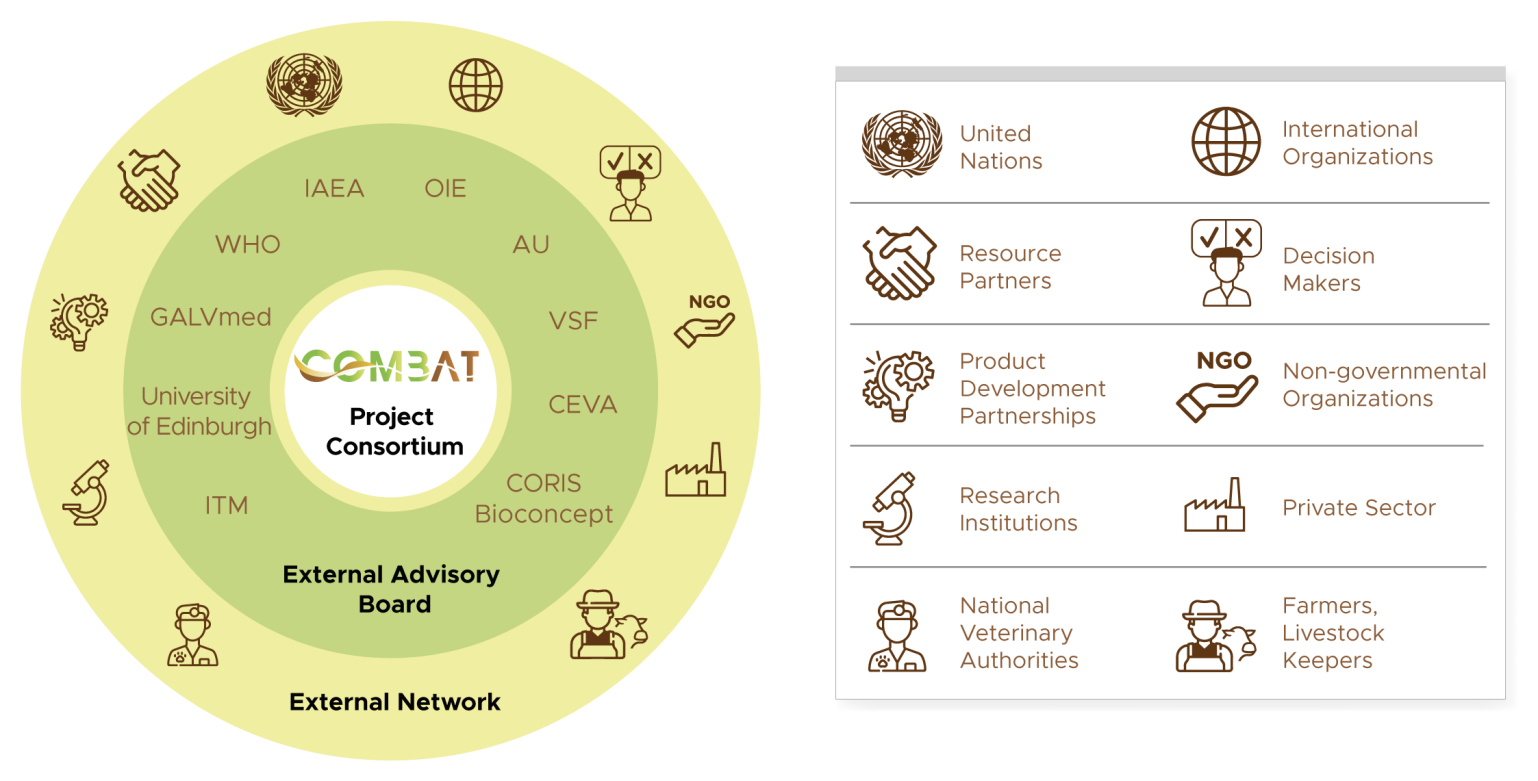
COMBAT external network and external advisory board. AU: African Union; GALVmed: Global Alliance for Livestock Veterinary Medicines; IAEA: International Atomic Energy Agency; ITM: Institute of Tropical Medicine Antwerp; OIE: World Organisation for Animal Health; VSF: Vétérinaires Sans Frontières (Veterinarians without borders); WHO: World Health Organization.

***External advisory board and external network.*** In addition to the large and diversified consortium, COMBAT will benefit from the external support of a broad network of stakeholders ( Figure 4). Some partners will be involved in the project as subcontractors, most notably in the case of private companies that produce specific tools. Others, such as decision-makers and resource partners, will be engaged through communication activities and participation in project events such as meetings and workshops. A selected group of external partners will be involved as advisors through an external advisory board (EAB), with a view towards reflecting in the project the perspectives of a wide range of actors. Prominent among the external advisors are the international organizations engaged in African trypanosomoses control and elimination. The AU will be engaged through its Pan-African Tsetse and Trypanosomiasis Eradication Campaign (PATTEC), and it will play an important role in raising awareness and in strengthening the commitment of decision-makers. WHO, OIE and IAEA will also participate in the EAB. This will allow them to contribute to, and benefit from, the project within their respective mandates and fields of expertise. Non-governmental organizations, farmers associations, other research institutions and both profit and not-for-profit organizations complete the network. External advisors and the broader external network will contribute to a wide dissemination of the project achievements and outputs, and to the long-term sustainability of its impacts.

In addition to the EAB, the project avails itself of other management structures, including a general assembly, an ethics advisory board and an exploitation committee.

## Project’s contribution to the sustainable development goals

COMBAT will improve knowledge of AT epidemiology, develop better control tools, strengthen disease surveillance and enhance capacities and engagement. However, the project’s impacts should be viewed in the broader context of the sustainable development goals (SDGs), in particular the elimination of poverty (SDG1), the ending of hunger (SDG2) and the promotion of good health and well-being (SDG3).

Increasing agricultural productivity is crucial for poverty reduction (SDG1), as agricultural workers constitute almost two-thirds of the extreme poor ( World Bank, 2018). By tackling trypanosomosis, COMBAT will contribute to SDG1 by boosting farmers’ income ( Bouyer *et al*., 2014). As an indication, the potential benefits from interventions against bovine trypanosomosis in East Africa were estimated at approximately USD 2.5 billion over a 20-year period, at an average of USD 3,300 per square kilometre of affected area ( Shaw *et al*., 2014).

More broadly for Africa, where 675 million people (52% of the population) are affected by moderate to severe food insecurity ( FAO *et al*., 2020), SDG2 will benefit from COMBAT in terms of reduction of disease burden and the related increase in livestock and crop productivity (e.g. increased meat and milk production, improved fertility, decreased mortality, improved draught power and organic fertilization of soils) ( Swallow, 2000). The potential for effective trypanosomosis control is illustrated by Kenya where, despite limitations in the control tools presently available, AT prevalence was reduced by 60% over a period of 15 years in high-priority areas ( Ngari *et al*., 2020). COMBAT will also improve food quality through enhanced livestock production and reduced contamination of meat by trypanocidal drugs and insecticides. The use of insecticides will be reduced by developing environmentally friendly vector control tools, and improved veterinary diagnostics will reduce unnecessary drug treatments by enabling test-and-treat strategies. In a context where tens of millions of doses of trypanocides are used annually in Africa, COMBAT will contribute to reducing AT incidence, thus decreasing the need for toxic products.

As to SDG3, the project will contribute to good health and well-being through new vector control tools and improved diagnostics, which will support the ongoing HAT elimination initiatives. At a time when the COVID-19 pandemic has drawn the world’s attention to zoonoses, the control of African trypanosomoses provides an example of the need for and potential of the One-Health approach.

While the project main impacts will be on SGDs 1, 2 and 3, COMBAT will also contribute to other SDGs. For example, increased income will benefit smallholder households, not least women and children (SDG 4 and 5). Increased livestock production will strengthen value-chains, for instance by boosting the dairy sector ( Swallow, 2000), creating employment in food industries (SDG8) and decreasing reliance on food imports. Healthier, disease-free animals can also contribute to climate change mitigation and adaptation (SDG13) ( FAO, 2020a), because AT-free animals have lower emissions intensity (i.e. lower emissions per unit of product) ( MacLeod *et al*., 2018), whilst at the same time they enhance the resilience of communities. COMBAT will also increase knowledge of local animal genetic resources and shed light on how certain livestock species and breeds cope better with AT ( FAO, 2020b). This will contribute to raising awareness of livestock biodiversity, inform the conservation of animal genetic resources (SDG 15), and provide data for the development of livestock genomics. Finally, with its large consortium of participants and broad external network, the project will contribute to SDG17 (partnerships for the goals).

## Conclusions

COMBAT was officially launched on 1 ^st^ September 2021, and it will run for four years. With 21 participants and an EU contribution of approximately 6 million Euros (€), it is to date the broadest project to fight vector-borne animal trypanosomosis in Africa. It is noteworthy that more than 50% of the EU contribution will directly benefit participating African institutions.

The project is expected to have a major impact on research and innovation, but further efforts and additional resources will be needed to translate these innovations into sustainable disease control.

For regular updates on project events and results, the COMBAT project website can be consulted. ( https://www.combat-project.eu).

## Abbreviations

**ARC:** Agricultural Research Council; **AT:** animal trypanosomosis; **AU:** African Union ; **CIRAD:** *Centre de Coopération Internationale en Recherche Agronomique pour le Développement*; **CIRDES:** *Centre International de Recherche Développement sur l’Elevage en zone Subhumide*; **COMBAT:** controlling and progressively minimizing the burden of animal trypanosomosis; **CVRL:** Central Veterinary Research Laboratory; **DIMS:** *Direction de l'Impact et du Marketing de la Science*; **DNA:** deoxyribonucleic acid; **EAB:** external advisory board; **FAO:** Food and Agriculture Organization of the United Nations; **GIS:** geographic information system; **GALVmed:** Global Alliance for Livestock Veterinary Medicines; **GPS:** global positioning system ; **HAT:** human African trypanosomosis; **IAEA:** International Atomic Energy Agency; **IBD:** *Insectarium de Bobo-Dioulasso*; **ICIPE:** International Centre of Insect Physiology and Ecology; **IES:** *Institut d'Electronique et des Systèmes*; **IPR:** *Institut Pierre Richet*; **IRD:** *Institut de Recherche pour le Développement*; **IRED:** *Institut de Recherche en Élevage pour le développement*; **ISRA:** *Institut Sénégalais de Recherches Agricoles*; **ITM:** Institute of Tropical Medicine Antwerp, **KENTTEC:** Kenya Tsetse and Trypanosomiasis Eradication Council; **LAMP:** loop-mediated isothermal amplification; **MAK:** Makerere University; **MSEG:** *Mission Spéciale d'Eradication des Glossines*; **NICETT:** National Institute for Control and Eradication of Tsetse fly and Trypanosomosis; **OIE:** World Organisation for Animal Health; **PAAT:** Programme Against African Trypanosomosis; **PATTEC:** Pan-African Tsetse and Trypanosomiasis Eradication Campaign; **PCP:** progressive control pathway; **PPR:** *peste des petits ruminants*; **RFID:** radio frequency identification; **SDG:** sustainable development goals; **SIT:** sterile insect technique; **T.:** *Trypanosoma*; **TCD:** Division of Tsetse Control services; **TTCU:** Tsetse and Trypanosomiasis Control Unit; **UEM:** *Universidade Eduardo Mondlane*; **ULPGC:** *Universidad de Las Palmas de Gran Canaria*; **VSF:** *Vétérinaires Sans Frontières*; **VUB:** *Vrije Universiteit Brussel*; **WHO:** World Health Organization.

## Data availability

No data are associated with this article.

## Ethics and consent statement

Ethical approval and consent were not required.

## Disclaimers

The boundaries and names shown, and the designations used on the maps presented in this paper do not imply the expression of any opinion whatsoever on the part of FAO concerning the legal status of any country, territory, city or area or of its authorities, or concerning the delimitation of its frontiers and boundaries. Dashed lines on maps represent approximate border lines for which there may not yet be full agreement. The final boundary between Sudan and South Sudan has not yet been determined.
