# Supplementary material for: The COMBAT project: controlling and progressively minimizing the burden of vector-borne animal trypanosomosis in Africa
Source: Open Res Eur. 2022 Aug 15;2:67. Originally published 2022 May 30. [Version 2] doi: 10.12688/openreseurope.14759.2 (PMC10445831; doi:10.12688/openreseurope.14759.2)
Supplement: Peer review history

# Peer review: Reviewer response for version 1

**DOI:** 10.21956/openreseurope.15941.r29415

**Published:** 2022-06-24

**Authors:** Jan Van Den Abbeele

**Affiliations:** 1 Department of Biomedical Sciences, Trypanosoma Unit, Institute of Tropical Medicine Antwerp, Antwerp, Belgium

COMBAT is a multidisciplinary and highly ambitious project on animal trypanosomosis (AT) with objectives ranging from advancing knowledge on various aspects of AT, to improvement and/or development of new control tools, up to capacity strengthening and impacting on the burden of AT in Africa. It is built upon a broad consortium having major stakeholders on board from different African AT-endemic countries and Europe. Improvements in overall veterinary health and more specifically in the control of AT at the small-holder farm level can be expected as major outcomes of this project, among others

 The text is well written giving a glimpse of the objectives and the broad spectrum of activities within this project.

Some minor comments :

- The authors state that the existing vector control tools are sub-optimal. It is not clear on which basis the authors make this claim. So far, insecticide-impregnated targets (combined with synthetic odour blends) and in some cases in combination with selective cattle insecticide spraying are shown to be highly effective in controlling tsetse of the morsitans group. Moreover, for palpalis group flies the insecticide-impregnated 'tiny targets' showed to be a highly effective vector control tool in the ongoing HAT-elimination activities.
- Typing error in Fig. 1, left pillar: environment.
- The one serological test (VeryDIAG) that is commercially available as a rapid diagnostic test for T *. congolense/T. vivax* is not mentioned in the text at all when presenting the development of diagnostic tools for AT. Is there any reason this is not mentioned?
- In several paragraphs it is not clear which trypanosomes are considered, e.g., when describing chemotherapy and drug resistance it is stated that COMBAT will collect trypanosomes, perform full genome sequencing, and annotation of both resistant and susceptible isolates, followed by comparative analysis. Is the aim to do this for all AT-causing trypanosomes ( *T. congolense, T. vivax , T. b. brucei, and T. evansi*) or will it be focused on specific trypanosome species? Please clarify. Also to be checked in other paragraphs. 
- The authors state that, as to SDG3, the project will contribute to good health and well-being through new vector control tools and improved diagnostics, which will support the ongoing HAT elimination initiatives. However, looking at the COMBAT activities, it is not clear how they will support the ongoing HAT-elimination initiatives. Currently, the latter is focused on *T.b. gambiense* and based upon serodiagnosis/parasitology + patient treatment combined with efficient tsetse fly control using tiny targets. Please reconsider your statement/this paragraph.
- It would be good to add a section that describes the major hurdles/difficulties that can be expected in such an overarching project and how they can be anticipated.

Where applicable, are recommendations and next steps explained clearly for others to follow? (Please consider whether others in the research community would be able to implement guidelines or recommendations and/or constructively engage in the debate)

Yes

Does the article adequately reference differing views and opinions?

Yes

Are all factual statements correct, and are statements and arguments made adequately supported by citations?

Yes

Is the rationale for the Open Letter provided in sufficient detail? (Please consider whether existing challenges in the field are outlined clearly and whether the purpose of the letter is explained)

Yes

Is the Open Letter written in accessible language? (Please consider whether all subject-specific terms, concepts and abbreviations are explained)

Yes

Reviewer Expertise:

Parasitology, African trypanosomosis

I confirm that I have read this submission and believe that I have an appropriate level of expertise to confirm that it is of an acceptable scientific standard.

# Response

**Published:** 2022-07-29

**Authors:** Alain Boulangé

**Affiliations:** CIRAD, France

Thanks for the positive feedback and the pertinent comments. We provide here below a point-by-point feedback on the minor comments raised by Prof Van Den Abbeele.

- The authors state that the existing vector control tools are sub-optimal. It is not clear on which basis the authors make this claim. So far, insecticide-impregnated targets (combined with synthetic odour blends) and in some cases in combination with selective cattle insecticide spraying are shown to be highly effective in controlling tsetse of the morsitans group. Moreover, for palpalis group flies the insecticide-impregnated 'tiny targets' showed to be a highly effective vector control tool in the ongoing HAT-elimination activities.

*Our statement is made in the context of existing challenges to sustainable, cost-effective control of animal trypanosomosis. We use the adjective “sub-optimal” in its most straightforward meaning of “less than optimal”/“less than ideal”, and by this we do not mean to claim that existing tools are “ineffective”. Indeed, we agree with the reviewer that several existing vector control tools are effective against the vectors of trypanosomosis. However, even effective tools have their shortcomings, and we believe that none of the existing ones can be considered “optimal” or “ideal”. We prefer not to go into the details of the strengths and weaknesses of each existing tool, which would be a lengthy and - in certain instances - controversial discussion. Suffice it to say that shortcomings in the existing tools may include (i) availability at the field level; (ii) ease of usage at the farmers’ level; (iii) cost; (iv) potential environmental impact (including challenges to safe disposal); (v) sustainability in case of long-term use; (vi) reliance on synthetic insecticides; (vii) heavy logistical requirements; and (vii) excessive reliance on external funding and donations. We believe that all existing vector control tools are affected by at least one of these shortcomings, and that therefore there is merit in efforts aimed at enhancing existing tools or developing new ones.* *That having been said, we fully agree with the reviewer that, because of the demonstrated effectiveness of some of the existing vector control tools, concerted efforts should be aimed at lifting the constraints to their use. Indeed, in the framework of the progressive control pathway for animal trypanosomosis, the optimization of usage of existing tools is considered a priority.* *As regards the topic of vector control to support ongoing HAT elimination activities, it is somewhat peripheral to the present paper and we prefer not to discuss it in more detail here. However, we added the recent reference below, which provides a continent-wide appraisal of vector control in the context of gambiense HAT elimination.*

- *“FAO and WHO: Vector control and the elimination of gambiense human African trypanosomiasis (HAT) - Joint FAO/WHO Virtual Expert Meeting - 5-6 October 2021. PAAT Meeting Report Series. No. 1. Food and Agriculture Organization of the United Nations and World Health Organization, Rome. 2022. https://doi.org/10.4060/cc0178en”*

*The reference was added in the section ‘Innovative control tools (pillar 2)’/‘Vector control and surveillance’, as:* *“Targets, and in particular tiny targets, are also used to support the elimination of gambiense HAT (FAO and WHO, 2022).” *

- Typing error in Fig. 1, left pillar: environment.

*We corrected this. Thanks.*

- The one serological test (VeryDIAG) that is commercially available as a rapid diagnostic test for T. congolense/T. vivax is not mentioned in the text at all when presenting the development of diagnostic tools for AT. Is there any reason this is not mentioned?

*There is a wide range of diagnostic methods for animal trypanosomoses, and the serological test VeryDIAG is just one of them, and not one of the most frequently used at that, and presents its own shortcomings. So, there is no particular reason why it is not mentioned in this paper. We just felt that it was not necessary to mention it specifically or discuss it.*

- In several paragraphs it is not clear which trypanosomes are considered, e.g., when describing chemotherapy and drug resistance it is stated that COMBAT will collect trypanosomes, perform full genome sequencing, and annotation of both resistant and susceptible isolates, followed by comparative analysis. Is the aim to do this for all AT causing trypanosomes (T. congolense, T. vivax , T. b. brucei, and T. evansi) or will it be focused on specific trypanosome species? Please clarify. Also to be checked in other paragraphs.

*We have added the requested details in the following paragraphs:* *Efforts will focus chiefly on* T. congolense *from West, East and Southern Africa, and to a lesser extent on *T. evansi *from Sudan, while other trypanosomes species may be considered.*  

- The authors state that, as to SDG3, the project will contribute to good health and well-being through new vector control tools and improved diagnostics, which will support the ongoing HAT elimination initiatives. However, looking at the COMBAT activities, it is not clear how they will support the ongoing HAT-elimination initiatives. Currently, the latter is focused on T.b. gambiense and based upon serodiagnosis/parasitology + patient treatment combined with efficient tsetse fly control using tiny targets. Please reconsider your statement/this paragraph.

*Ongoing HAT elimination initiatives spearheaded by WHO are not limited to the elimination of transmission of *T. b. gambiense *. Infection with *T. b. rhodesiense *is included in the WHO road map for neglected tropical diseases 2021–2030, with the target of “elimination as a public health problem”. To achieve the goals set for rhodesiense sleeping sickness, WHO calls for effective surveillance, early detection and early response to *T. b. rhodesiense *cases, including possible outbreaks. In response to this call, COMBAT will develop innovative diagnostics through novel antigen detection tests that can be used to detect T. b. rhodesiense in humans.* *Regarding vector control, it is true that tiny targets in the past few years the most widely used vector control tool in the areas affected by gambiense HAT, and that they have been effective in contributing to reduction of gambiense HAT transmission. However, as already mentioned earlier in the context of animal trypanosomosis control, even tiny targets have their shortcomings, and vector control in rhodesiense HAT area should also not be neglected. We believe that COMBAT activities aimed at developing novel vector control tools have the potential to contribute to SDG3 in a One-health framework (e.g. by developing new insecticide-free, biodegradable devices). *

- It would be good to add a section that describes the major hurdles/difficulties that can be expected in such an overarching project and how they can be anticipated.

*As similar point was raise by reviewer Brice Rotureau, and we agree that this addition is useful. Therefore, we added the following paragraph to the ‘Conclusions’ section.* *“Moreover, the project will have to manage a number of risks and challenges, including the coordination of a large and diverse consortium, limitations to travel and in-person events imposed by the COVID-19 pandemic, the spike in commodity prices, and the insecurity and instability that affect some of the areas and countries where COMBAT operates.”* *We take the opportunity to mention that the COMBAT project avails itself of a Risk management plan that is regularly updated.*

# Peer review: Reviewer response for version 1

**DOI:** 10.21956/openreseurope.15941.r29413

**Published:** 2022-06-24

**Authors:** Harriet Auty

**Affiliations:** 1 Institute of Biodiversity, Animal Health & Comparative Medicine, College of Medical, Veterinary & Life Sciences, University of Glasgow, Glasgow, UK

This letter introduces and describes a large collaborative project focused on improving control of animal African trypanosomiasis (AAT). The introduction provides an accurate description of the challenges of improving control and highlights the limited progress in contrast to human African trypanosomiasis. Factors limiting progress are identified, including the role of wildlife reservoirs of disease, lack of appropriate diagnostics, outdated trypanocidal drugs, and lack of data on socioeconomic impacts. It is essential that these issues are tackled to make progress on sustainable AAT control, and it is very encouraging to see such investment in this area.

 The paper describes four themes of work which make up the COMBAT project. Brief descriptions of the work in each area are included. The range of work covered in the topics is broad, with numerous specific projects listed within each section. Although not likely addressable given the word limits, this does limit how much can be understood about the specific questions to be tackled and approaches planned in each of the work areas.

 The project includes a range of stakeholders, including research organisations in Europe and Africa, and national veterinary authorities in affected countries, nicely described in Figure 2. This collaborative approach, particularly including FAO as a partner and involving national veterinary authorities, is certainly a strength, and should provide a strong base for implementing change. The paper describes how the project is built around the Progressive Control Pathway (PCP) framework. One of the outputs will be guidelines to inform the implementation of the PCP, which is likely to be a helpful route to actually achieve improved control.

 It is not very clear how the findings from different and quite diverse work areas will be integrated to ensure that a comprehensive step forward in AAT control is achieved, or what mechanisms are in place to share findings between the multiple and relevant partners, in order to develop new guidelines. With the large number of partners involved, this is likely to be challenging, and presumably plans exist to achieve this.

 In a couple of places in the paper, ‘a low level of awareness of decision-makers, donors and national veterinary authorities’ is mentioned. Although there is no doubt that a lack of political will is a factor limiting better control, this may not always stem from a lack of awareness. These factors are discussed in more detail elsewhere in the paper, but this phrasing could be nuanced to reflect that.

 Overall this paper describes an exciting project and a significant and much needed investment in improving AAT control. If the team are able to achieve what they have described, this will be remarkable progress in developing and implementing tools for AAT research and control.

Where applicable, are recommendations and next steps explained clearly for others to follow? (Please consider whether others in the research community would be able to implement guidelines or recommendations and/or constructively engage in the debate)

Yes

Does the article adequately reference differing views and opinions?

Yes

Are all factual statements correct, and are statements and arguments made adequately supported by citations?

Yes

Is the rationale for the Open Letter provided in sufficient detail? (Please consider whether existing challenges in the field are outlined clearly and whether the purpose of the letter is explained)

Yes

Is the Open Letter written in accessible language? (Please consider whether all subject-specific terms, concepts and abbreviations are explained)

Yes

Reviewer Expertise:

Veterinary epidemiology, epidemiology of trypanosomiasis

I confirm that I have read this submission and believe that I have an appropriate level of expertise to confirm that it is of an acceptable scientific standard.

# Response

**Published:** 2022-07-29

**Authors:** Alain Boulangé

**Affiliations:** CIRAD, France

*Thanks for the positive feedback and the pertinent comments. We provide here feedback on the two points raised by Dr Auty.*

- It is not very clear how the findings from different and quite diverse work areas will be integrated to ensure that a comprehensive step forward in AAT control is achieved, or what mechanisms are in place to share findings between the multiple and relevant partners, in order to develop new guidelines. With the large number of partners involved, this is likely to be challenging, and presumably plans exist to achieve this.

*The point raised by the reviewer is valid. We provide here some elements of clarification, but we prefer not to modify the body of the paper to discuss these issues.* *One of the main strategies for the integration of the diverse areas of work rests on the fact that most project participating institutions and project countries are involved in several project pillars and work packages. In particular, several COMBAT institutions work on both research and innovation (pillar 1 and 2) and on risk-based control strategies (pillar 3). This will facilitate the update of innovative tools and improved knowledge in the national policies and practices for diseases control.* *Pillar 4, which includes capacity development, will also play a key role in project integration. A range of training activities and initiatives will allow project participants as well as external stakeholders to acquire the necessary skills to apply the innovative tools. Communication and dissemination of project results, first within, and then outside the project consortium, will also be actively pursued.* *Finally, the ‘Exploitation committee’, established as one of the project management structures, will also contribute to integration by addressing issues related to intellectual property, data management as well as commercial and non-commercial exploitation of project results. *

- In a couple of places in the paper, ‘a low level of awareness of decision-makers, donors and national veterinary authorities’ is mentioned. Although there is no doubt that a lack of political will is a factor limiting better control, this may not always stem from a lack of awareness. These factors are discussed in more detail elsewhere in the paper, but this phrasing could be nuanced to reflect that.

*We agree with the reviewer on the fact that a lack of political will is not always stemming from a lack of awareness, but we think that our references to the issue of awareness are always made in connection with other causes and issues, and as such they may not need further nuancing.*

# Peer review: Reviewer response for version 1

**DOI:** 10.21956/openreseurope.15941.r29414

**Published:** 2022-06-14

**Authors:** Brice Rotureau

**Affiliations:** 1 Trypanosome Transmission Group, Trypanosome Cell Biology Unit and INSERM, Institut Pasteur, Paris, France; 2 Parasitology Unit, Institut Pasteur of Guinea, Conakry, Guinea

Controlling and progressively minimizing the burden of animal trypanosomosis (COMBAT) is a four-year research and innovation project funded by the European Commission, whose ultimate goal is to reduce the burden of animal trypanosomosis (AT) in Africa. This open letter successively presents the background, primary and secondary objectives, organization, and project’s contribution to the sustainable development goals. It is very well written and offers a clear and simple overview of this project.  

 The COMBAT project builds on the progressive control pathway (PCP), a risk-based, step-wise approach to disease reduction or elimination. The COMBAT project is structured into four thematic pillars: (1) improved knowledge of AT epidemiology (i.e. vector-parasite-host-environment interactions), (2) development of innovative control tools, (3) disease risk mapping, surveillance and evidence-based control strategies, and (4) reinforced capacities and engagement of stakeholders. Data collection, analysis and storage for evidence-based decision making underpin all COMBAT activities. The project consortium comprises 21 participants, including a geographically balanced representation from 13 African countries, and it will engage a larger number of AT-affected countries through regional initiatives.

 This ambitious project is just impressive. It smartly covers a wide range of multidisciplinary activities dwelling with almost all the components ruling AT epidemiology. COMBAT will undoubtedly lead to major improvements in veterinary health and the generated knowledge and partner framework will be key for controlling AT in the future.  

 Minor comments:

- As the open letter format is limited, it is frustrating not to have a more in-depth description of the WP, especially for pillars 1 and 2.
- A short paragraph describing the most important expected challenges and the main possible difficulties that would mitigate the project feasibility would be appreciated in a risk assessment section.
- In Fig2, the use of symbols coloured according to the groups of partners they represent would be a plus.    

Where applicable, are recommendations and next steps explained clearly for others to follow? (Please consider whether others in the research community would be able to implement guidelines or recommendations and/or constructively engage in the debate)

Yes

Does the article adequately reference differing views and opinions?

Yes

Are all factual statements correct, and are statements and arguments made adequately supported by citations?

Yes

Is the rationale for the Open Letter provided in sufficient detail? (Please consider whether existing challenges in the field are outlined clearly and whether the purpose of the letter is explained)

Yes

Is the Open Letter written in accessible language? (Please consider whether all subject-specific terms, concepts and abbreviations are explained)

Yes

Reviewer Expertise:

Parasitology

I confirm that I have read this submission and believe that I have an appropriate level of expertise to confirm that it is of an acceptable scientific standard.

# Response

**Published:** 2022-07-29

**Authors:** Alain Boulangé

**Affiliations:** CIRAD, France

- As the open letter format is limited, it is frustrating not to have a more in-depth description of the WP, especially for pillars 1 and 2.

*We agree that more in-depth descriptions of the WPs would be interesting, but, as understood by the reviewer, the format of the paper does not lend itself to this. Further details on a range of project activities will be disseminated in future publications.*

- A short paragraph describing the most important expected challenges and the main possible difficulties that would mitigate the project feasibility would be appreciated in a risk assessment section.

*We agree that this is a useful addition, and, as mentioned in the similar comment made by Prof Van den Abbeele, we have added the following paragraph:* *“Moreover, the project will have to manage a number of risks and challenges, including the coordination of a large and diverse consortium, limitations to travel and in-person events imposed by the COVID-19 pandemic, the spike in commodity prices, and the insecurity and instability that affect some of the areas and countries where COMBAT operates.”*

- In Fig2, the use of symbols coloured according to the groups of partners they represent would be a plus.

*We see the reviewer’s point here. However, colours are already used in figure 2 to mark both the geographical location (dashed rectangles) and the focus of activity (coloured circles) of the institutions involved. We fear that using also “symbols coloured according to the groups of partners” could complicate rather than facilitate the reading and interpretation of the figure and the colours thereof, given that some project partners belong to more than one group, so we prefer to keep the symbols colours as they are.*
